# Toward TiO_2_ Nanofluids—Part 1: Preparation and Properties

**DOI:** 10.1186/s11671-017-2184-8

**Published:** 2017-06-15

**Authors:** Liu Yang, Yuhan Hu

**Affiliations:** 1 Key Laboratory of Energy Thermal Conversion and Control of Ministry of Education, School of Energy and Environment, Southeast University, Nanjing, China; 2 Jiangsu Provincial Key Laboratory of Solar Energy Science and Technology, School of Energy and Environment, Southeast University, Nanjing, China

**Keywords:** Nanofluids, Preparation, Stability, Property, Surface tension

## Abstract

As a new generation of working fluid, nanofluid has long been regarded as a hot research topic in the past three decades. Many review papers have provided comprehensive and systematic summaries on the development and state-of-the-art of nanofluids. As of today, it is becoming increasingly difficult to provide a comprehensive review of all kinds of nanofluids owing to the huge amounts of the related literatures. And many controversies and inconsistencies in the reported arguments have been observed in various nanofluids. Meanwhile, the systematic or comprehensive reviews on a certain kind of nanofluid are insufficient. Therefore, this review focuses on the research about one of the hottest kinds viz. TiO_2_ nanofluid, which has captured scientists’ great attention because of its interesting and comprehensive properties such as sensational dispersivity, chemical stability, and non-toxicity. Due to the preparation of nanofluids is the prerequisite and physical properties are critical factors for further applications, this first part of the review summarizes recent research on preparation, stability, and physical properties of TiO_2_ nanofluids.

## Review

### Background

#### Development of Nanofluids

Since the heat transfer capacity of liquids is generally far below that of solid metals or metal compounds, it is expected that the heat transport of liquid can be enhanced by suspending solid particles into it. However, some drawbacks appeared in suspensions with millimeter or micrometer particles, such as the poor dispersibility, aggregation, and sedimentation as well as adhering to inner surface of the system, which could easily lead to degradation of heat transfer performance, increases in pumping power, and even pipe block. A new opportunity to overcome these drawbacks was found when a new generation of suspension viz. nanofluid was proposed by Choi in 1995 [1].

Nanofluid is a new kind of dilute suspension containing nanoparticles whose at least one-dimensional size is below 100 nm. When the particle sizes in the suspension reach nanometer level, it is expected that the suspension can achieve a better thermal property and simultaneously keep more stable than millimeter or micrometer particles/liquid mixture. A stable nanofluid can also obtain a better liquidity, and sometimes, it can be treated as single-phase fluid. Therefore, one of the biggest challenges nanofluids face is the preparation and stability, which are the principal prerequisite for achieving good thermophysical properties and further engineering applications. Accordingly, the research on the nanofluids can generally be categorized into the following directions: preparation and stability study [2, 3], physical properties such as thermal conductivity [4–8] and viscosity analysis [9–12], heat transfer research [13, 14], engineering application [15–18], and theoretical analysis or model development [19–25].

**Fig. 1:**
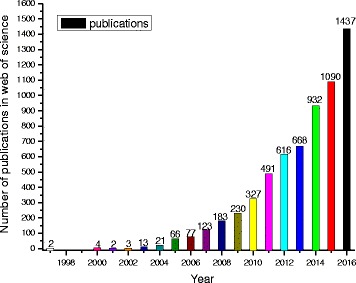
Number of publications containing “nanofluids or nanofluid” in title retrieved from “web of science”

In the past two decades especially recent 10 years, the research about nanofluids has been explosively increasing due to their fascinating properties and many researchers have conducted the experimental or theoretical studies on various aspects of nanofluids [26–29]. To illustrate this, the growth trend in the number of publications containing “nanofluids or nanofluid” in title retrieved from “web of science” can be found in Fig. 1. This figure clearly illustrates that the research of nanofluids is growing so fast that the publication in 2016 has landed 21.9% of the total in the past two decades. If the retrieval scope was relaxed to full text and to contain more search databases, the results could increase several times. Therefore, it is becoming increasingly difficult to provide a comprehensive review of all kinds of nanofluids owing to the huge amounts of the related literatures. And in the last 2 years, a few reviews have focused on one aspect of property or a certain kind of nanofluid to provide more comprehensive reviews. For instance, Table 1 shows the latest reviews on some specialized aspects of nanofluids such as:

1. Preparation or characterization [30–32]
2. Certain kinds of nanoparticles (Al_2_O_3_, TiO_2_, CuO, graphene, CNT, hybrid nanofluids) [32–38]
3. Certain kinds of base fluid (water, EG, EG/water mixture, oil) [39–42]
4. One or more physical properties (thermal conductivity, viscosity, specific heat) [43–47]
5. Certain kinds of characteristics (forced, nature, boiling convection heat transfer, pressure drop, particle migration) [48–53]
6. Some specialized applications (heat exchanger, solar collectors, refrigeration) [54–62]

**Table 1:** Summary of the latest reviews on some specialized aspects of nanofluids

Researchers	Year	The aspect of reviews focusing on	Classification
Kong et al. [30]	2017	Preparation, characterization, and tribological mechanism	Preparation and characterization
Sharma et al. [31]	2016	Preparation and evaluation of stable nanofluids	Preparation and characterization
Yazid et al. [32]	2016	Preparation on stability of carbon nanotube nanofluids	Preparation and characterization, particle type: CNT nanofluids
Sundar et al. [33]	2017	Preparation, thermal properties, heat transfer, and friction factor of hybrid nanofluids	Particle type: hybrid nanofluids
Khurana et al. [34]	2017	Forced convection heat transfer and pressure drop of Al_2_O_3_, TiO_2_, and CuO nanofluids	Particle type: Al_2_O_3_, TiO_2_, and CuO nanofluids
Yang et al. [35]	2017	Heat transfer of TiO_2_ nanofluids	Particle type: TiO_2_ nanofluids
Sidik et al. [36]	2016	Hybrid nanofluids in heat transfer applications	Particle type: hybrid nanofluids
Rasheed et al. [37]	2016	Graphene-based nanofluids	Particle type: graphene nanofluids
Sadeghinezhad et al. [38]	2016	Graphene nanofluids	Particle type: graphene nanofluids
Akilu et al. [39]	2016	Thermophysical properties of water-based composite nanofluids	Base fluid type: water
Murshed et al. [40]	2016	Conduction and convection heat transfer characteristics of ethylene glycol-based nanofluids	Base fluid type: ethylene glycol
Azmi et al. [41]	2016	Heat transfer augmentation of ethylene glycol: water nanofluids and applications	Base fluid type: ethylene glycol/water mixture
Rafiq et al. [42]	2015	Properties of transformer oil-based nanofluids	Base fluid type: transformer Oil
Mukherjee et al. [43]	2016	Role of temperature on thermal conductivity of nanofluids	Physical property: thermal conductivity
Bashirnezhad et al. [44]	2016	Experimental studies of viscosity of nanofluids	Physical property: viscosity
Azmi et al. [45]	2016	Thermal conductivity and dynamic viscosity of nanofluids	Physical property: thermal conductivity and viscosity
Aybar et al. [46]	2015	Thermal conductivity models	Physical property: thermal conductivity models
Sharma et al. [47]	2016	Rheological behavior of nanofluids	Physical property: rheological behavior
Bahiraei et al. [48]	2016	Particle migration in nanofluids	Characteristics: particle migration in nanofluids
Pinto et al. [49]	2016	Heat transfer enhancement mechanisms	Characteristics: heat transfer mechanisms
Singh and Gupta [50]	2016	Heat transfer in a tube under constant heat flux boundary condition	Characteristics: heat transfer in tube for constant heat flux
Fang et al. [51]	2016	Heat transfer and critical heat flux of nanofluid boiling	Characteristics: boiling heat transfer
Huminic and Huminic [52]	2016	Heat transfer and flow characteristics in curved tubes	Characteristics: heat transfer and flow in curved tubes
Vanaki et al. [53]	2016	Numerical study of convective heat transfer	Characteristics: convective heat transfer
Cai et al. [133]	2017	Fractal-based approaches in aggregation	Research method: fractal method
Verma et al. [54]	2017	Application in solar collectors	Application: solar collectors
Kasaeian et al. [55]	2017	Flow and heat transfer in porous media	Application: porous media
M’hamed et al. [56]	2016	External magnetic field on nanofluids	Application: coupled with magnetic field
Muhammad et al. [57]	2016	Thermal performance of stationary solar collectors	Application: solar collectors
Khond and Kriplani [58]	2016	Performances and emissions of emulsified diesel and biodiesel fueled stationary CI engine	Application: stationary CI engine
Hussien et al. [59]	2016	Single-phase heat transfer enhancement in micro/minichannels	Application: micro/minichannels
Patil et al. [60]	2015	Thermo-physical properties and performance characteristics of a refrigeration system	Application: refrigeration
Sarsam et al. [61]	2015	Nanofluids in flat-plate solar collectors	Application: solar collectors
Kumar et al. [62]	2015	Nanofluids in plate heat exchanger	Application: plate heat exchanger

### Advantages of TiO_2_ Nanofluids

**Fig. 2:**
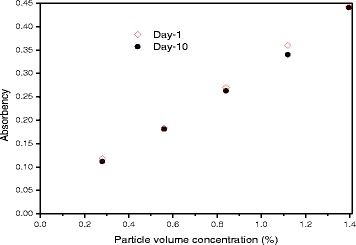
Absorbance as a function of particle volume concentration day 1 and day 10 [66]. Reproduced with permission from Elsevier

The above introductions in Table 1 exhibit the feasibility and significance of reviews on some specialized directions of nanofluids since it can provide relatively comprehensive and detailed information for a certain aspect. As one of the most prevalent kinds, TiO_2_ nanofluids have captured scientists’ great attention due to their excellent physical and chemical properties. Firstly, TiO_2_ is widely used in the fields of printing, cosmetics, air purification, etc., and it is a universally recognized safe material without any toxicity for human beings. Considering the safety of this nanofluid, Taghizadeh-Tabari et al. [63] have applied TiO_2_–water nanofluid in a plate heat exchanger for milk pasteurization industries. Secondly, TiO_2_ has exceptional chemical stability, resistance to acid, alkali, and most organic solution erosion. Thirdly, TiO_2_ nanoparticles have been produced in larger industrial grade which makes them relatively economical [64]. Fourthly, TiO_2_ nanoparticles have relatively good dispensability in both polar and nonpolar base fluids especially when adding proper dispersant. Yang et al. [65] investigated the dispersion stabilities of 20 types of nanoparticles in ammonia–water solution. The results showed that anatase TiO_2_ was the most stable metal oxide without surfactant, and its stability could be further improved by adding proper surfactant. In Silambarasan et al.’s report [66], the absorbency of TiO_2_ nanofluids varied very little after 10 days’ storage as shown in Fig. 2. Such slight change in absorbency indicates that the stability of TiO_2_ nanofluids they prepared was fairly remarkable. It can be concluded by summarizing available literatures that TiO_2_ nanoparticles, in general, have a better dispensability than other conventional metal oxide nanoparticles. Since the dispersion of nanoparticles in liquid is the most important prerequisite for application of nanofluids, many researchers have selected TiO_2_ nanofluids as research subjects.

**Fig. 3:**
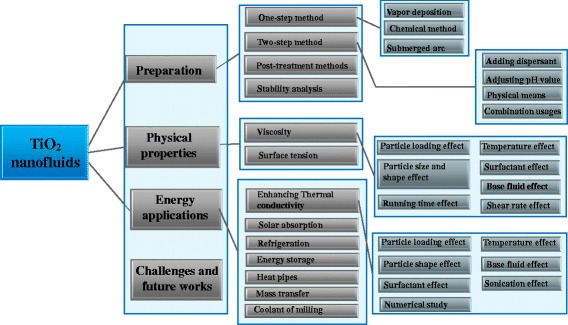
A diagrammatic sketch of the preparation, properties, applications, and challenges of TiO_2_ nanofluids

Due to the preparation of nanofluids is the prerequisite and physical properties are critical factors for designing and building the energy related applications, the aim of the two reviews is to systematically summarize the recent study progresses on TiO_2_ nanofluids, including the preparation, stability, physical properties, and energy applications. A detailed diagrammatic sketch of the two reviews on the preparation, property, and application of TiO_2_ nanofluids can be seen in Fig. 3. This review is organized from the perspective of a certain kind of nanofluid, which is considered as one of the closest kinds to the practical application. And the main goal of this paper is to provide a helpful reference guide for researchers to update the knowledge on research status of TiO_2_ nanofluids and point out the critical challenges and useful recommendations for future study directions.

## Preparation of TiO_2_ Nanofluids

### One-Step Method

Generally, two main preparation methods can be differentiated: one-step and two-step methods. One-step method is implemented by suspending nanoparticles into required working fluid accompanying their generation process. One-step method can be further subdivided into physical methods and chemical methods. Physical method includes vapor deposition, laser ablation, and submerged arc. Chemical method means to produce nanofluids by chemical reaction. Generally, the above methods are introduced as the preparation methods of dry nanoparticles. However, those methods can be upgraded to one-step preparation methods of nanofluids by replacing the dry particle collectors to the corresponding base fluid containers.

#### Vapor Deposition

**Fig. 4:**
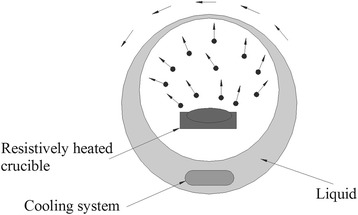
A typical device of vapor deposition method for the preparation of nanofluids. Redrawn based on reference [67]

Vapor deposition is a common physical method in the preparation of nanofluid. A typical device for this method can be viewed in Fig. 4 [67]. The bulk solid material for preparing nanoparticles is heated and evaporated in a low-pressure container filled with an inert gas, and then, the vapor of raw material is cooled by the swirling liquid film and settled in the base fluids. The vapor deposition is usually used in the preparation of metal nanofluids, but this method is rarely employed for TiO_2_ nanofluids because of the high temperature of the boiling point. However, this method can be improved by using electric heating to achieve a high temperature. Lee et al. [68] used a one-step pulsed wire evaporation (PWE) method to prepare ethylene glycol (EG)-based nanofluids containing TiO_2_ nanoparticles. They applied pulsed 25-kV voltages across a thin wire and overheated it to evaporate into plasma in a few milliseconds. Then, the plasma was interacted by argon oxygen and condensed into nanoparticles. Finally, they obtained TiO_2_ nanofluids by letting the nanoparticles directly contact EG inside the chamber wall.

#### Submerged Arc Method

**Fig. 5:**
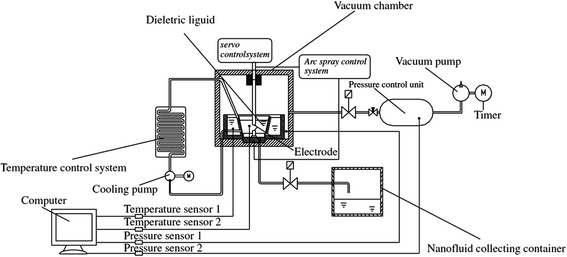
Schematic fig. of the improved submerged arc nanofluid synthesis system (ISANSS) [40]. Reproduced with permission from The Japan Institute of Metals and Materials

The submerged arc method can provide and keep an even higher temperature for the preparation of TiO_2_ nanofluids. Chang et al. [69] manufactured a new submerged arc synthesis system to produce TiO_2_ nanofluids. Their device is mainly composed of arc spray unit, vacuum space, and temperature and pressure control systems, which is shown in Fig. 5. In this device, bulk TiO_2_ solid was vaporized by the arc discharge method in a vacuum, and then, the gaseous TiO_2_ was cooled rapidly into fine solid by an isolated liquid. They concluded that this method was more prominent than aerosol methods because the prepared nanofluids had higher dispersion stability and could be considered as a Newtonian fluid. Zhang et al. [70] improved the submerged arc method by optimizing the reaction parameter control system, cooling circulation and the size of the submerged arc device. Based on the optimized system, they can produce more stable and finer TiO_2_ suspension with good reproducibility in particle size. And the adsorption performance of their TiO_2_ nanoparticles is better than commercial ones.

#### Chemical Method

Chemical method is to obtain nanofluids by chemical reaction, and it generally includes coprecipitation method and precursor conversion method. The conventional chemical method of synthesizing TiO_2_ nanofluids is based on a precursor TiO(OH)_2_ sediment by chemical reaction of titanic inorganic salts and ammonia–water, then undergo calcination to obtain TiO_2_ powder. Some research showed that nanofluids obtained by the chemical method had better stability and higher thermal conductivity than those produced by the two-step method [71]. The controllability of the microstructure of nanoparticles is another distinguishing feature of this method. The conventional adjusting method is to control the parameters such as the synthesis temperature, pH value, ultrasonic bath time, and the additives [72]. However, this method is mainly used to prepare TiO_2_ powder by drying the liquid as a result of the complex liquid environment in this method is not suitable for the detailed application of nanofluids. While when the TiO_2_ powders can stably suspend in the required base fluid by changing the bulk fluid without drying process, this method will be promising under the condition that the new liquid environment parameters such as acidity or alkalinity and electrolyte concentration are close to original fluid for preparation.

One-step method has not contained the drying and dispersing processes which are vulnerable to form agglomeration of nanoparticles. Therefore, one-step method is generally believed to obtain more stable nanofluids [73]. However, there are also some defects restrict the application range of the one-step method. For example, vapor deposition cannot be utilized to prepare the nanofluids containing high boiling point or non-crystal nanoparticles. Laser ablation and vacuum buried arc methods are in high-cost and require critical circumstance conditions. The chemical method generally requires the services of specific reaction conditions such as required pH value and temperature. And it can easily synthesize some by-products in the liquids [74]. For example, Sonawane et al. [75] used sol–gel method to synthesize anatase TiO_2_ nanoparticles with a constant pH value of 5. The precursor solution included titanium isopropoxide and isopropanol as well as double-distilled water. It can be concluded that this mixture with such specific pH value and complex chemical compositions could not be used as the heat transfer nanofluids. Therefore, they dried the synthesized TiO_2_ nanoparticles and then re-dispersed them into required base working fluids including water, EG, and paraffin oil with ultrasonic treatments to obtain the required nanofluids. It can be concluded that the one-step method is hardly to be utilized for some nanofluids with specific ingredients, especially for the nanofluids with pure water, oil, refrigerant, etc. as base fluids and also for an application system containing volatile gas.

### Two-Step Method

**Fig. 6:**
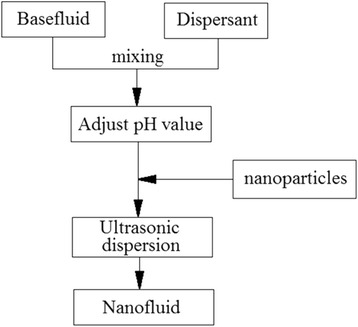
A typical procedure of two-step method of preparation of nanofluids [35]. Reproduced with permission from Elsevier

In two-step method, the processes of producing nanoparticles and suspending them into required base fluid are operated independently. Two-step method is widely used for TiO_2_ nanofluids since the synthesis techniques of TiO_2_ nanoparticles have essentially reached the industrial production scale. Figure 6 displays a typical procedure of two-step method. The dry nanoparticles are firstly synthesized by chemical or physical methods and then suspended into required base fluids. However, because the strong particle interaction force might lead to colliding and aggregating of nanoparticles, it is rather difficult for them to suspend stably and uniformly in the base fluid. Therefore, some dispersion methods are employed in general to ensure a good stability and availability of nanofluids.

Table 2 shows a summary of related studies on the preparation methods of TiO_2_ nanofluids in recent years. It can be seen that the types of base fluid have involved water, EG, refrigerant, organic solvents, etc. In general, three main techniques for the dispersion and suspending of nanoparticles in base fluids were widely utilized in the two-step preparation process.

**Table 2:** Summary of related studies on the preparation methods of TiO_2_ nanofluids in recent years

Researchers	Base fluid	Particle size (nm)	TiO_2_ loading	Dispersant	pH	Physical means	Stable time
Mo et al. [77]	Water	20 × 50, 15	0.05–0.7 wt.%	SDS	8	Ultrasonication	286 h
Mo et al. [78]	Water	15	0.7 wt.%	SDBS, PVP, CTAB	8	Sonication + stirring	3 days
Fedele et al. [89]	Water	72–76	1–35 wt.%	Acetic acid	1.86–3.07	Sonication	35 days
Liu et al. [88]	Water	25	3 wt.%	PEG1000	4–5, 9–10	Ultrasound vibration	168 h
Kim et al. [91]	Water, EG	10/34/70	1–3 vol.%	SDS	–	Sonication + stirring	–
Mushed et al. [92]	Water	15, 10 × 40	0–0.55 vol.%	Oleic acid, CTAB	6.8–6.2		–
Jarahnejad et al. [93]	Water	30	3–9 wt.%	Polycarboxylate, trioxadecane acid	7.2, 7.5		–
Ghadimi et al. [90]	Water	25	0.1 wt.%	SDS	5	Ultrasonic vibration	1 year
Said et al. [94]	Water	21	0.1–0.3 vol.%	PEG400	9	High-pressure homogenizer	30 days
Murshed et al. [134]	EG	15, 10 × 40	1–5 vol.%	CTAB		Ultrasonication	–
Reddy et al. [135]	W, W + EG (6:4) W + EG (1:1)	40	0.2–1 vol.%	Oleic acid and CTAB		Ultrasonic bath	–
Saleh et al. [109]	Water	33	0.05–5 vol.%	CTAB, SDS, span 80		Sonication + stirring	–
Peng et al. [96]	R141b	25, 40, 60, 100	25–500 mg/L	SDBS, CTAB, NP-10		Ultrasonication	–
Nakayama and Hayashi [79]	Organic solvents	3.2	10 wt.%	Hexanoic acid, n-hexylamine	–	Sonication	
Wu et al. [95]	Ammonia–water	15	0.1 vol.%	PAA	–	Sonication + stirring	48 h
Yang et al. [74]	Ammonia–water	15	1–4vol.%	PEG1000		Sonication + stirring	–
Duangthongsuk and Wongwises [136]	Water	21	0.2 vol.%	CTAB	–	Ultrasonic vibration	–
Srinivas et al. [137]	Water	10	0.3–2 wt.%	CTAB	–	Ultrasonic vibration	–
Megatif et al. [138]	Water	27	0.1–0.2 wt.%	SDBS	–	Sonication	–
Tazarv et al. [132]	R141b	30	0.01–0.03 vol.%	CTAB	–	Ultrasonication + stirring	1 week
Bobbo et al. [120]	Water	21	0.01–1 wt.%	PEG800	–	High-pressure homogenizer	18 days
Kayhani et al. [97]	Water	15	0.1–2 vol.%	HMDS		Ultrasonic vibration	Several days
Li and Sun [80]	SRFA and Fe(III)	30	50 mg/L		4, 6, 8	Sonication	A few days
Setia et al. [139]	Water	–	0.5–0.75 vol.%		3	Ultrasonication	–
He et al. [81]	Water	20	0.24–1.18 vol.%	–	11	Ultrasonication	Months
Hu et al. [140]	Water	10	0.94–2.78 vol.%	–	8	–	–
Yiamsawasd et al. [141]	Water, W + EG (8:2)	21	0–8 vol.% (W) 0–4 vol.% (W + EG)	–	7 (W), 6 (W + Eg)	Ultrasonic vibrator	–
Longo et al. [142]	EG	15	1–3 vol.%	None	8.3	Sonication + stirring	Ensure test period
Chakraborty et al. [143]	Water	20 × 100	0.1–2 wt.%	None	6.5	Ultrasonic vibrator	–
Pak and Cho [144]	Water	27	1–10 vol.%	None	10	Stirring	5–6 days
Vakili et al. [82]	Water	25	0.5–1.5 vol.%	None	11	Ultrasonic vibrator	24 h
Muthusamy et al. [84]	EG	30–50	0.5–1.5 vol.%	None	8.2–8.5	Mechanical stirring	>3 weeks
Sen et al. [83]	Aqueous electrolytes	25	20–50 wt.%	None	11	Ultrasonic bath	>1 month
Trisaksri et al. [145]	R141b	21	0.01–0.05 vol.%	–	–	Ultrasonic vibration	3–4 weeks
Padmanabhan et al. [101]	R134a and mineral oil	–	0.1 g/L	–	–	Magnetic stirrer	6 months
Tajik et al. [86]	Water	30–40	0.005–0.2 vol.%	None	–	Ultrasonic pulses	48 h
Longo et al. [87]	Water	30–50	1–6 vol.%	None	–	Sonication + stirring	>1 month
Tavman et al. [146]	Water	21	0.2–3 vol.%	None	–	Ultrasonication	–
Wang et al. [147]	EG	40	0–4 vol.%	None	–	Ultrasonic bath	–
Palabiyik et al. [102]	Propylene glycol	21	0.25–2.4	None	–	Sonication	Several months
Zhang et al. [148]	Water	40	0–2.6 vol.%	None	–	Sonication	48 h
Lokwani et al. [149]	Water	25	0.25–1 wt.%	–	–	Ultrasonication	30 days
Li et al. [85]	MDEA	15	0.05–0.8 wt.%	None	–	Ultrasonication	48 h
Sajadi and Kazemi [150]	Water	30	0.02–0.25 vol.%	None	–	Ultrasonication	–
Leena et al. [151]	Water	15	0.04–0.2 wt.%	None	–	Ultrasonication	4–6 days
Mostafizur et al. [152]	Methanol	21	0.01–0.15 vol.%	None	–	Ultrasonication	7 days
Sonawane et al. [75]	Water/EG/paraffin oil	5	1–6 vol.%	None	–	Ultrasonication	A few hours

#### Adding Dispersant

The first dispersion method is modifying the particle surfaces by adding dispersant, which is expected to prevent the nanoparticles from aggregating by the roles of electrostatic repulsion or steric hindrance of the dispersant molecules [76]. It can be noted that the most frequently employed surfactant was CTAB in the existing reports. And other kinds including SDBS, SDS, PVP, oleic acid, acetic acid, and PEG were also used on some research. In 2012, Mo et al. [77] used two-step method to prepare two kinds of nanofluids by suspending rod-like rutile TiO_2_ and spherical anatase TiO_2_ into water. They observed that the nanofluids can keep stable for 286 h when using SDS as dispersant. In the following year, they compared the effects on dispersion by three different surfactants including SDBS, PVP, and CTAB [78]. And they found that in this experimental research scope, when the mass ratio of the SDBS and the titanium dioxide nanoparticles is 0.3, they can get the best dispersion of nanofluid. Nakayama and Hayashi [79] used two-step method to disperse a high loading of TiO_2_ nanoparticles in an organic base liquid with the help of surface modification by propionic acid and n-hexylamine. They found the surface modification can improve the dispersion of nanofluids, which showed better effect on two-step method than on one-step method. The characteristics of TiO_2_ nanoparticles they prepared are not changed, and they can be well applied for different organic solvent base fluids.

#### Adjusting pH Value

The second dispersion method is to adjust the dispersion environment by adjusting the pH value of the base fluid. This method is to equip the nanoparticles higher zeta potential by adjusting a suitable pH value of fluid, which is expected to avoid the contacting of nanoparticles by the higher electrostatic repulsions [76]. Li and Sun [80] investigated the effect of pH value on aggregation behaviors of TiO_2_ nanoparticles in mono- and binary base liquids by SRFA and Fe(III). They found that the adsorption of SRFA greatly improved the suspending stability of TiO_2_ nanoparticles at pH values of 4, 6, and 8, and they thought that this mainly caused by the sharp rise of negative charges on the particles’ surface. He et al. [81] found that the stability of TiO_2_ nanofluids can be greatly improved by adjusting the pH value of the base fluid to 11, at which a high zeta potential of 45 mV can be formed to prevent re-agglomeration and deposition and possible subsequent fouling the copper tube. The nanofluids with the optimal pH value can keep stable for several months. Also, Vakili et al. [82] and Sen et al. [83] adjusted the pH value of the base fluid to 11, and they found that the TiO_2_ nanofluids can have better dispersion stability under this strongly alkaline condition.

#### Physical Means

The third dispersion method is tantamount to breaking particle agglomerations by physical means, for instance mechanical agitation, ultrasonic waves, and stirred bead milling. Those methods are supposed to generate cavitation oscillations which can lead to shearing, breaking, and dispersing effects [84]. It is universally recognized and proved that the nanofluids will be more stable after proper supersonic vibration and it can be proved once again by the summary of the dispersion stability TiO_2_ nanofluids. It can be seen from Table 2 that almost all the preparation processes have involved some physical treatments. Li et al. [85] dispersed TiO_2_ nanoparticles into MDEA solution to prepare TiO_2_–MEDA–H_2_O nanofluids. They found that the nanofluids could keep stable for 48 h with mechanical agitation without adding dispersant. Tajik et al. [86] investigated the effects of different ultrasonic types (continuous or discontinues pulses) on the suspending behavior of water-based TiO_2_ nanofluids. The results showed that the continuous pulses had better breaking effects than the discontinuous ones, while the latter could not separate some big aggregations. Silambarasan et al. [66] investigated experimentally the effect of stirred bead milling and ultrasonication on the suspending behavior of water-based mixture containing submicron TiO_2_ particles. They found that stirred bead milling can produce stable suspensions containing submicron particles, and ultrasonication can be further applied to control the transport behavior of the TiO_2_ suspensions. Longo and Zilio [87] compared the effects of mechanical stirring and ultrasonic waves on the dispersion behavior of TiO_2_–water and Al_2_O_3_–water nanofluids. They observed that treatment of sonicating at 25 kHz for 48 h showed better dispersion efficiency than just simple mechanical stirring. After these physical dispersion treatments, the both kinds of nanofluids can keep stable for more than 1 month.

#### Combination Usages

Generally, combinations of dispersion methods of adding surfactant, changing pH value of base fluids, and ultrasound vibration are utilized in two-step method to achieve better dispersion performance of nanofluids. Liu et al. [88] dispersed TiO_2_ nanoparticles (25 nm) in water to prepare stable TiO_2_ nanofluids. Three treatments including addition of PEG1000 as dispersant, ultrasound vibration, and regulating the pH value to 4–5 or 9–10 were utilized to obtain stable TiO_2_ nanofluids. Fedele et al. [89] used a combination dispersion method of adding acetic acid as dispersant and adjusting pH value to a range from 1.86 to 3.07 according to the mass fractions of nanoparticles as well as a suitable sonication; they observed that the nanofluids could keep stable for at least 35 days because the mean sizes of particles remained approximately constant during the periods. Ghadimi et al. [90] prepared an extremely stable water-based TiO_2_ nanofluid by adding acetic acid and adjusting pH to 5 as well as ultrasonic vibration. They found the TiO_2_ nanofluids were still stably suspended after 1 year of storage. There are also some other examples for the combined use of the three techniques. It can be found form Table 2 that Mo et al. [77, 78], Kim et al. [91], Mushed et al. [92], Jarahnejad et al. [93], Ghadimi et al. [90], and Said et al. [94] utilized all of the three dispersion techniques to achieve the best dispersion effect.

However, changing pH value of base fluids will severely restrict the application range of the TiO_2_ nanofluids as thermal fluids due to the corrosion and safety in acidic and alkaline conditions. Therefore, more researchers are more inclined to employ the other two dispersion techniques viz. adding dispersant and physical means for the potential applications in actual systems. Wu et al. [95] and Yang et al. [74] intended to apply TiO_2_ nanofluids to ammonia–water absorption refrigeration system. The method of changing the pH value is not available because the base fluid has a specific pH range determined by the concentration of ammonia. Therefore, they used PAA or PEG1000 as dispersant combined with ultrasonic vibration to improve the stability of TiO_2_ nanofluids and achieved good effects. To apply nanofluids to compression refrigeration system, Peng et al. [96] added TiO_2_ nanoparticles into R141b to prepare nano-refrigerant with particle size of 25, 40, 60, and 100 nm respectively. The nano-refrigerant was sonicated using an ultrasonic processor for 20 min. And they thought this step is important to achieve good dispersion for nanoparticles in bulk refrigerant. Also, they studied experimentally the influence of anionic, cationic, and nonionic surfactants on the stability of nano-refrigerant. And they observed that the surfactant type is an important factor on the steady-state particle size. Kayhani et al. [97] used surfactant hexamethyldisilazane and ultrasonic vibration methods prepared dry TiO_2_ nanoparticles firstly and then added into distilled water with ultrasonic vibration (400 W and 24 kHz) treatment for 3–5 h. They found that the prepared nanofluids could keep stable for several days and no sedimentation occurred. Yang et al. [98] found that the usages of surfactant SDBS at a low-concentration range and ultrasonic vibration can improve the suspending behavior of ammonia-water based TiO_2_ nanofluids.

### Post-treatment Methods

Besides conventional one-step or two-step method, some post-treatment methods for the preparation of nanofluids were also proposed. Some better dispersed nanofluids may be obtained from some poorly dispersed raw fluids containing agglomerated nanoparticles through some special treatments, such as break down or remove the agglomerated nanoparticles from the raw fluid.

**Fig. 7:**
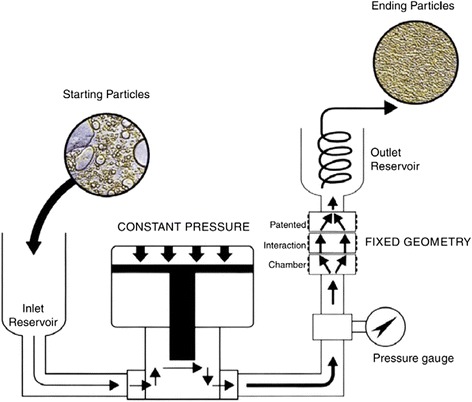
Schematic diagram of the high-pressure homogenizer for producing nanofluids [99]. Reproduced with permission from Elsevier

Hwang et al. [99] observed that the effects of stirrer, ultrasonic bath, and ultrasonic disrupter are limited for improving the dispersion of nanofluids. They used a high-pressure homogenizer to retreat the nanofluid, and the process can be seen in Fig. 7. In their research, the initial average diameter of the particles can be decreased by at least one order of magnitude after the re-treatment by the high-pressure homogenizer. And they found that the high-pressure homogenizer exhibited the best effect among all the physical dispersion means used in their study.

**Fig. 8:**
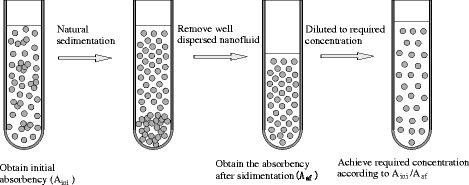
Optimizing process of dispersion improvement of nanofluids [132]. Reproduced with permission from Taylor & Francis

Yang et al. [100] used an optimizing method to prepare nanofluids. The optimizing process of dispersion improvement of nanofluids is shown in Fig. 8. They removed the well-suspended nanofluids from the bulk higher concentrated nanofluids and then regained the removed parts into the required concentrations by dilution of adding base fluids. The dilution ratio was based on the property if absorbency of the nanofluids is directly proportional to its concentration. And they observed sedimentations and measured the varying of absorbency to estimate the effect of the method. The results showed that for both rutile and anatase TiO_2_ nanofluids, the optimized method can greatly improve their dispersion and produce more stable TiO_2_ nanofluids.

There are some controversies or inconsistencies in arguments of preparation of nanofluids. Firstly, whether to adopt the one-step method or two-step method is an inconsistency. One-step method is expected to achieve better dispersion stability since it avoids the drying and dispersing processes of nanoparticles. However, for the side effects of the one-step method such as by-product, special solution environment seem more fatal which severely restricts the application scope of nanofluids. Therefore, two-step method is more widely used due to the great adaptability and substantial improvement in the dispersion techniques of TiO_2_ nanoparticles. Overall, two-step method is recommended to be employed with appropriate post-treatment for the preparation of TiO_2_ nanofluids.

Another controversy is whether surfactant should be used in the preparation of nanofluids. The presence of appropriate surfactant can improve the dispersion stability but also may bring some side effects such as a decrease in thermal conductivity, increases in viscosity, and foaming ability. Due to the potential advantages such as reduction in surface tension and improvement in re-dispersible property, the surfactant with low concentration is suggested to be used when it not brings obvious decrease in thermal conductivity or increase in viscosity and foaming ability. In addition, the influence of surfactant on thermal conductivity and viscosity of nanofluids is also a controversy in current studies.

## Stability of Nanofluids

Stability research is generally followed the preparation to achieve the optimal dispersion craft since it is closely related to the effectiveness and practicability of nanofluids. The great amount of aggregations in the unstable nanofluids can easily cause sedimentation and adsorption on the inner surface of the system, which will probably result in the degradation of heat transfer efficiency, raising of pumping power, and even blocking up in system pipe blocks.

It can be found from Table 2 that the stable times of different researchers thought were variously distributed in the range of several hours to 1 year. A most stable nanofluid was obtained by a combined use of adding surfactant, controlling pH value, and ultrasonic vibration by Ghadimi et al. [90]. Also, the particles’ loading in their experiment was very low at 0.1 wt.%, which was also another contribution for the long-term stability. Without adding surfactant, the nanofluids can also achieve a better dispersion stability by adjusting the pH value of the liquid to a suitable value. For example, He et al. [81] and Longo et al. [87] observed that the TiO_2_ nanofluids can keep stable for months by adjusting the pH to 11 with the help of ultrasonic vibration. Also, some TiO_2_ nanofluids with good dispersion stability were prepared only through physical means in some research. Padmanabhan et al. [101] used a magnetic stirring to prepare R134a and mineral oil-based TiO_2_ nanofluids that can keep stable for 6 months. This is likely because the particles’ loading employed in their study is very low (0.1 g/L) and the high viscosity of the R134a and mineral oil base fluid can also provide a superior dispersion condition. This conclusion can also serve as proved by Palabiyik et al.’s results [102]. They obtained a TiO_2_ nanofluids stable for several months by the help of sonication with a higher viscosity propylene glycol as base fluid. The similarity is that they were both using organic solvent of high viscosity as base fluids and the best ones was only treated by physical means. Also, it can be seen that TiO_2_ nanoparticles have a comprehensive dispersivity in both polar aqueous solution and nonpolar organic solution.

However, the above judgments on dispersion stability of various TiO_2_ nanofluids are not very objective and accurate because most of the results showed the least stable time. Moreover, there is no uniform standard for evaluating the stability of nanofluids, and the stability evaluating methods in different research were sufficiently different. Current evaluation methods of stability of nanofluids mostly consisted of observing the stratification or sedimentation and testing the zeta potential, particles’ size, or absorbency. Mansel et al. [103] used the sedimentation observation method and zeta potential method to evaluate the stability of TiO_2_–water nanofluids in different pH values. They observed that in low or high pH value, the TiO_2_–water nanofluids can obtain good stability. Mo et al. [78] used zeta potential method to investigate the stability of TiO_2_–water nanofluids with three different surfactants SDBS, PVP, and CTAB, respectively. By comparing the value of zeta potential, they obtained the optimal kind of surfactant and the best dispersion of nanofluid. Wei et al. [104] used sedimentation, zeta potential (ζ), and size analysis to evaluate the stability of diathermic oil-based TiO_2_ nanofluids. They found that there was not obvious sedimentation and the zeta potential (ζ) and size analysis also showed good results. They thought the TiO_2_ nanofluids they prepared were very stable and can be used to enhance heat transfer for a fluid system. Li et al. [105] used sedimentation observation to investigate the stability of TiO_2_–MDEA–water nanofluids. They found that after a specific period of mechanical agitation, the sedimentation was reduced and the stability of nanofluids was improved. However, the ultrasonic vibration will deteriorate the stability of TiO_2_–MDEA–water nanofluids. For this reason, only mechanical agitation was employed in their research. Yang et al. [74] investigated the dispersion behavior of 20 types of nanoparticles in binary base fluid of ammonia–water by measuring the absorbency of nanofluids, and they defined ratio of varying absorbency to quantitatively compare the suspending stability of different kinds of nanoparticles, dispersant, and base fluid mixtures. They observed that the new defined index was more applicable than conventional means because it could directly compare the suspending behavior of various kinds of nanofluids. While the method of observing the stratification or sedimentation is restricted for nanofluids in different colors or without distinctly stratification after standing. The results showed that the anatase and rutile TiO_2_ nanofluid were the most stable metal oxides without any surfactant. And when adding optimal dispersant, anatase TiO_2_ nanofluid was still the most stable one.

Generally, the combination of several stability evaluating methods is employed to investigate the stability of nanofluids more accurately. Silambarasan et al. [66] used method of measuring the particle size distribution, zeta potential, and microscopy of grain size methods to characterize the suspending stability of TiO_2_ nanofluids. By those methods, they prepared remarkably stable TiO_2_ nanofluids whose absorbency changed very little after 10 days. Tajik et al. [86] used sedimentation observation and microscopy of grain size to investigate the roles of ultrasonic wave types on the suspending behavior of nanofluids. And they found that the pulses in discontinues type could not smash some big clusters or aggregations since the sedimentation occurred after 48 h of storage.

## Physical Properties of TiO_2_ Nanofluids

The physical properties of TiO_2_ nanofluids are focused on the viscosity and thermal conductivity. Also, a few papers investigated the surface tension. Using nanofluids to enhance the thermal conductivity is a typical application in heat transfer filed. Therefore, the thermal conductivity of TiO_2_ nanofluids will be introduced in part 2 of the reviews. In part 1, the viscosity and surface tension are introduced as follows.

### Viscosity

Viscosity is an essential parameter for nanofluids especially for flow and heat transfer applications because both the pressure drop and the resulting pumping power are depended on the viscosity. Viscosity describes the internal resistance of a fluid to flow, and it is an important property for all thermal and flow applications for nanofluids. The nanofluids with higher viscosity will result in higher flow resistance and lower flow velocity, which also induce the decrease of the heat transfer. To obtain flow velocity and heat transfer efficiency, more pumping powers are needed which induce more energy consumption. Moreover, for some mass transfer application of nanofluids, viscosity plays more important roles than thermal conductivity because the viscosity determines the mass transfer resistance of molecules entering the liquid surface and the diffusion coefficient in the liquid. Murshed and Estellé [106] provide a state-of-the-art review on the viscosity of various nanofluids. They found that the experimental data from various literatures are greatly scattered and not consistent even for the same nanofluids. This review will discuss in detail the influence factors on the viscosity of TiO_2_ nanofluids to provide an exhaustive knowledge on this topic.

#### Particle Loading Effect

Many literatures have concerned the volume concentration effect on the viscosity of TiO_2_ nanofluids. Table 3 shows the particle loading dependence of the viscosity of TiO_2_ nanofluids in different research. It can be observed that the viscosity of the TiO_2_ nanofluids increases with the increase of the particle loading. However, some works showed that the viscosity ratio varies linearly with variation of volume concentration, but some other results showed the viscosity ratio variation is parabolic. The viscosity enhancements of TiO_2_ nanofluids were greatly distinguishing in various researches. For example, in Vakili et al. [82], Arulprakasajothi et al. [107], Duangthongsuk and Wongwises [108], Saleh et al. [109], and Mahbubul et al.’s [110] results, the increments of viscosity were below ten times of the volume percentage of the added particles. However, He et al. [111] and Turgut et al.’s [112] results showed that the viscosities were increased by more than 100 times of the volume percentage of the TiO_2_ particles added. There are also many results distributed between the values in the above two extreme cases. Therefore, it can be concluded that the influence of particle loading on the viscosity of TiO_2_ nanofluids is more complex than that on thermal conductivity due to the widespread data in various studies.

**Table 3:** Particle loading dependence of the viscosity of TiO_2_ nanofluids in different research

Researchers	Base fluid	Particle shape	Particle size (nm)	Volume fraction	Viscosity increment (%)	Whether Newtonian fluids
He et al. [111]	Water	Spherical	20	0.125–1%	11–141	Yes
	Water	Spherical	20	0.125–1%	15–141	Yes
He and Zheng [153]	BaCl2–water	Spherical	–	0.167–1.13%	2.86–31.9	Yes
Ling et al. [113]	Water	Spherical	35	0–0.225%	0–7.15	–
Chen et al. [64]	EG	Rod-like	10 × 100	0–1.8%	0–72	No
	Water	Rod-like	10 × 100	0.1–0.6%	1–82	No
	EG	Spherical	25	0.25–1.2%	3–11	Yes
	Water	Spherical	25	0.1–1.86%	0.5–23	No
Duangthongsuk and Wongwises [108]	Water	Spherical	21	0.2–2%	4–15	–
Mahbubul et al. [110]	R123	Spherical	21	0.5–2%	1.3–5.2	–
Yiamsawas et al. [123]	EG/water (20/80 wt.%)	Spherical	21	1–4%	13.6–60	–
Saleh et al. [109]	Water	Spherical	33	0.05–5%	1–40	–
Yiamsawas et al. [115]	Water	Spherical	21	1–8%	10–125	–
Turgut et al. [112]	Water	Spherical	21	0.2–3%	4–135	–
Arulprakasajothi et al. [107]	Water	Spherical	32	0.1–0.75%	0.5–2.1	Yes
Murshed et al. [154]	Water	Spherical	15	1–5%	25–82	–
Masuda et al. [155]	Water	Spherical	27	1–5%	10–82	–
Lokwani1 et al. [149]	Water	Spherical	25	0.25–1%	68–84	–
Pak and Cho [144]	Water	Spherical	27	1–10%	2.5–200	No
Bobbo et al. [120]	Water	Spherical	21	0.01–1 wt.%	−2.29 to 6.87	Yes
Vakili et al. [82]	Water	Spherical	25	0.5–1.5%	2–5.03	–
Sen et al. [83]	Aqueous electrolytes	Spherical	25	0–20 wt.%	0–380	–
Yapici et al. [118]	PEG200	Spherical	21	5 wt.%	15–108	No

#### Temperature Effect

Besides the volume concentration effect, the temperature effect on the viscosity of TiO_2_ nanofluids is also widely studied by many researchers. He et al. [111] prepared four different concentration TiO_2_–H_2_O nanofluids with 20 nm TiO_2_ and measured the viscosities of TiO_2_–H_2_O nanofluids and deionized water with different temperatures. They observed that the TiO_2_–H_2_O nanofluids were Newtonian fluids, which were the same as Chang and Liu’s finding [69], and the viscosities varied inversely with the temperature of the TiO_2_–H_2_O mixture system. Ling et al. [113] also measured the viscosities of the TiO_2_–H_2_O nanofluids with different mass fractions, when temperature varied from 15 to 40 °C. They found that the viscosity of the nanofluids increased when fluids thicken and decreased with the increment of the temperature exponentially. Liu et al. [114] figured that the viscosities of TiO_2_–H_2_O nanofluids increase remarkably with the volume fraction of nanoparticles and vary oppositely to the temperature of the TiO_2_–H_2_O nanofluids greatly with similar experimental method. Based on the value of the viscosities, they also propose an amended suspension viscosity formula. Some research results showed that the viscosity of nanofluids is a function of volume loading and temperature as well as base fluid viscosity. Yiamsawas et al. [115] measured the viscosity of TiO_2_–water with a volume loading varied from 1 to 8% at a high-temperature range of 15 to 60 °C. By comparisons with previous studies, they proposed a useful correlation for practical applications which indicated that the viscosity of nanofluids is a function of volume loading and temperature as well as the base fluid’s viscosity.

**Fig. 9:**
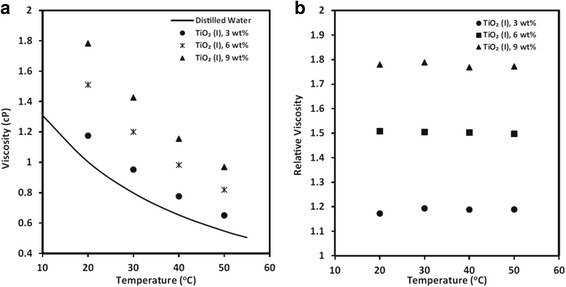
Dynamic viscosity (**a**) and relative viscosity (**b**) for TiO_2_ water-based nanofluids at different temperatures [93]. Reproduced with permission from Springer

Comparing with the absolute viscosity, the varieties of relative viscosity at different temperatures were more impressive for researchers. Jarahnejad et al. [93] carried out a detailed study on the effect of temperature on the viscosity and the relative viscosity of TiO_2_ respectively. And the results are shown in Fig. 9. It can be found that compared to base water, the average viscosities of TiO_2_ nanofluids increased by 17, 50, and 78% for 3, 6, and 9 wt.% of particles’ loading, respectively, at 20 °C. The viscosity of nanofluids with different particle loading decreased as the temperature increased, while the relative viscosity remained nearly constant with the temperature. The observation of independent of temperature can be also included in some other research. Fedele et al. [89] presented the characterization of water-based nanofluids where TiO_2_ ranging between 1 and 35% in mass. They concluded that the relative viscosity was independent from temperature for all the particle loading employed. And the nanofluids at 1 wt.% exhibited a water-like behavior within the experimental error. But this observation was invalid at the higher concentrations (+243% for 35 wt.% at 343 K). Also, Silambarasan et al. [66] found that the temperature has a smaller effect on the relative viscosity since the viscosity of TiO_2_ suspensions was reproducible even after repeated and alternating heating and cooling processes. And they attributed the reason to the effect of particles’ temperature-dependent intermolecular forces in the suspension. However, some different results can also be observed. Teng et al. [116] found that the relative viscosity increased from 8.2 to 16% when the temperature varied from 10 to 40 °C for the TiO_2_ nanofluids with 0.5 wt.% of particle loading. Cieśliński et al. [117] found that the relative viscosity of thermal oil-based TiO_2_ nanofluids remained constant when the temperature varied from 20 to 40 °C, but had a nearly linear increase with the increase of temperature when exceeding 40 °C. Yapici et al. [118] observed that the effect temperature was different for different shear rate. The relative viscosity measured was independent of the temperature at a higher shear rate region. However, for lower shear rate region, a great temperature dependency behavior of viscosity of TiO_2_ nanofluids was exhibited especially at high temperatures

#### Particle Size and Shape Effect

The particle size and shape effects on the viscosity of TiO_2_ nanofluids were not investigated as widely as that of particles’ loading or temperature. In particular, Chen et al. [64, 119] investigated experimentally the viscosity of spherical (25 nm) and rod-like (10 × 100) TiO_2_ nanoparticle-based nanofluids with water and EG as base fluid, respectively. They found that the viscosity of TiO_2_ nanofluids was more sensitive to the rod-like particles than spherical particles. It can be seen from Table 3 that the viscosity was increased by 0.5–23% when adding 0.1–1.86 vol.% of spherical TiO_2_ nanoparticles, while increased by 1–82% when adding 0.1–0.6 vol.% of rod-like TiO_2_ nanoparticles. The same observation can also be found for EG-based nanofluids.

#### Surfactant Effect

**Fig. 10:**
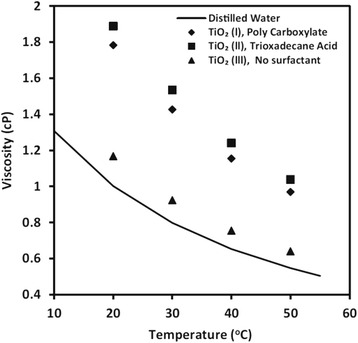
Dynamic viscosity of 9 wt.% TiO_2_–water nanofluids with different surfactants vs. temperature [93]. Reproduced with permission from Springer

The surfactants have been observed to have great effects on the viscosity of TiO_2_ nanofluids in some recent research. Jarahnejad et al. [93] investigated the effect of two kinds of surfactant trioxadecane acid and poly carboxylate on the viscosity of TiO_2_ nanofluids respectively. Their results of the dynamic viscosity of 9 wt.% TiO_2_–water nanofluids with different surfactants vs. temperature are shown in Fig. 10. The results demonstrated only a very slight increase was found in the viscosity of nanofluids even with the highest particle loading viz. 9 wt.%. However, the two kinds of surfactants could greatly increase the viscosity of nanofluids in the temperature range of 20–50 °C, especially for trioxadecane acid. The similar effect of surfactant on viscosity can also be observed in Ghadimi and Metselaar’s report [90], in which they found SDS can also increase the viscosity of TiO_2_ nanofluids with 0.1 wt.% particle loading. It was also observed there were important roles of SDS in the long-term dispersion stability of TiO_2_ nanofluids. Therefore, they still suggested that the dispersion method of adding surfactant and ultrasonic vibration to be adopted in the preparation of nanofluids.

**Fig. 11:**
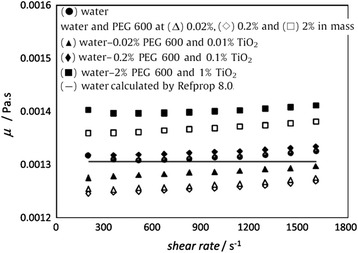
The viscosity of TiO_2_ nanofluids with PEG600 as surfactant [120]. Reproduced with permission from Elsevier

However, the above results cannot prove that all kinds of surfactant will result in high viscosity for nanofluids. Figure 11 shows the viscosity of TiO_2_ nanofluids with PEG600 as surfactant measured by Bobbo et al. [120]. It can be seen that the viscosity of base water will not increase but decrease slightly when adding PEG600 at 0.02 or 0.2% loadings. Also, the viscosity of nanofluid containing 0.01% TiO_2_ nanoparticles and 0.02% PEG600 was a little lower than that of the base water. However, for higher loading of PEG, the viscosity will be greatly increased whether or not containing nanoparticles. It can be seen from Fig. 11 that the nanofluids containing 2% PEG600 and 1% TiO_2_ nanoparticles showed a viscosity higher than 7% in respect to water, which was analogous at each temperature. The above observation showed the viscosity of nanofluids can be lower than the base fluid in some cases, which also occurred in SWCNT nanofluids in their experiment. The decline of viscosity of fluid when adding surfactant or nanoparticles was also been found in some other research. Yang et al. [121] found that emulsifier OP-10 can reduce the viscosity of ammonia–water in lower concentrations. Ling et al. [122] observed that adding SDBS or OP-10 in TiO_2_ nanofluids with a lower loading can induce a slight drop in viscosity. Therefore, it is an important issue to choose the suitable surfactants to improve the dispersion stability without increasing the viscosity significantly.

#### Base Fluid Effect

The information about base fluid effect on viscosity can be illuminated though Chen et al.’s study [119], in which they found the relative increments of viscosity of water-based TiO_2_ nanofluids were distinctly higher than that of EG based. It seemed that the higher viscosity the base fluid could result in lower increment in viscosity. Mahbubul et al. [110] found that the viscosity of R123 was increased by only 5.2% when adding 2 vol.% TiO_2_ nanoparticles. Sen et al. [78] and Yapici et al. [118] found relative increments of viscosity about 20 times of the particles’ volume percentages. It also seems that TiO_2_ nanoparticles are more suitable in the organic liquid because a lower relative increment in viscosity can be obtained especially at the higher temperature. Yiamsawas et al. [123] conducted experiments on a mixture with TiO_2_ nanoparticles and EG/water (20/80 wt.%) in which the volume loading ranged from 0 to 4% and temperature ranged from 15 and 60 °C. They used the experimental data to present a useful correlation to predict the viscosity.

#### Shear Rate Effect

**Fig. 12:**
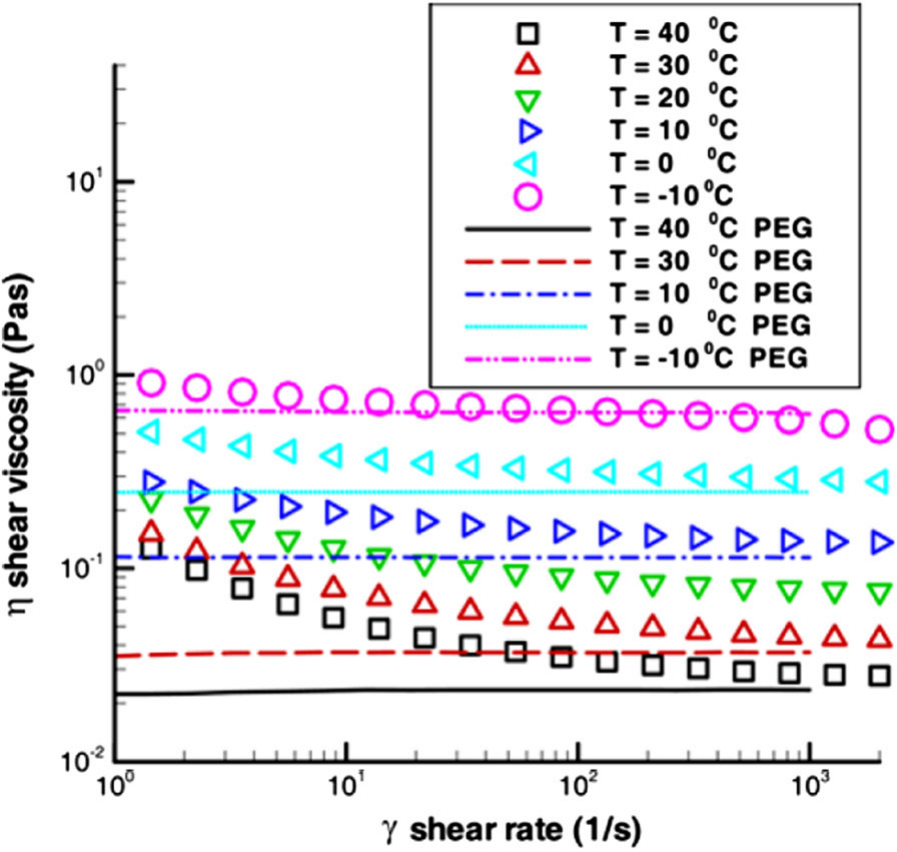
Shear rate dependency of viscosity as a function of temperature for 5 wt.% TiO_2_–PEG200 nanofluids [118]. Reproduced with permission from Springer

Another main distinction on the viscosity of TiO_2_ nanofluids in different research is that whether the fluids were Newtonian fluids in different shear rates. A typical Newtonian nanofluid can be found in foregoing Fig. 11. However, it can be observed from Table 3 that more than half of the results showed that the TiO_2_ nanofluids in their work are Newtonian fluids, but some others come to the opposite conclusion. Research on rheological characteristic has demonstrated that whether or not the TiO_2_ nanofluids exhibit Newtonian behavior is also affected by other factors, including the base fluid type, temperature, and particle loading. A quintessential example can be found in Chen et al.’s research [64], where they measured the viscosity of four types of nanofluids made of TiO_2_ nanoparticles (25 nm) and TiO_2_ nanotubes (10 nm × 100 nm) dispersed in water and EG. They found that EG–TiO_2_ nanofluids exhibited Newtonian behavior, whereas water–TiO_2_, water–TNT, and EG–TNT nanofluids exhibited non-Newtonian behavior. They indicated that the rheology behavior of TiO_2_ nanofluids is affected by their specific ingredient and environment, such as particles’ shape and liquid circumstance. The rheological characteristic of TiO_2_ nanofluids is also related to the temperature. Yapici et al. [118] investigated the rheological characteristic of 9 wt.% TiO_2_–water nanofluids with different surfactants vs. temperature. The results are shown in Fig. 12. It can be observed that the base fluid PEG was a typical Newtonian fluid in all kinds of temperature. However, TiO_2_–PEG200 nanofluids were nearly Newtonian fluid at a lower temperature and higher shear rate, but it changed into non-Newtonian fluid at higher temperature and lower shear rates. Also, in Said et al.’s results [94], the TiO_2_ nanofluid with 0.1 vol.% loading was Newtonian fluid at 55 °C, whereas it was non-Newtonian below this temperature for 0.3 vol.% particle loading.

#### Running Time Effect

**Fig. 13:**
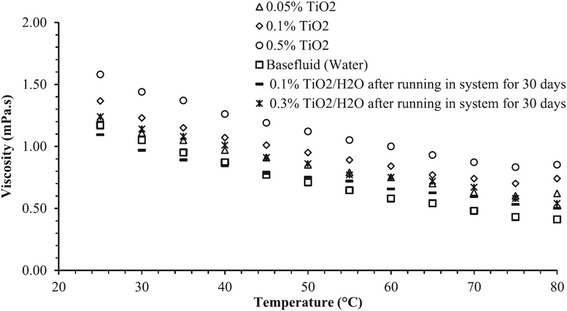
Viscosity of TiO_2_–water nanofluid with different volume concentrations and different temperatures [94]. Reproduced with permission from Elsevier

When the nanofluids are actually used in a running system, the time-dependent properties of nanofluids should be a crucial issue for the sustainable application. However, this matter has not been widely studied because of the faultiness in the development of nanofluids. It is generally considered that the thermal and rheological properties of nanofluids will be deteriorated due to the aggregation of nanoparticles after running a long time in the system. However, an opposite result in the time-dependent viscosity of TiO_2_ nanofluids can be observed in Said et al.’s research [94]. Their results for viscosity of TiO_2_–water nanofluid with different volume loading and temperature as well as running time are shown in Fig. 13. It can be observed that the viscosity of fresh samples and the stale samples after running in a flat plate solar collector for 1 month were distinctly different. The viscosity of TiO_2_ nanofluids was decreased after undergoing the alternative variations in temperature and flow rate in the cycle. This observation was quite interesting and could not be explained anywhere else in the literature. They thought this finding could open new research scope for the applications of nanofluids for a long-term use.

An inconsistency in viscosity of TiO_2_ nanofluids is quite evident. The intensities of growth in viscosity of TiO_2_ nanofluids with particle loadings greatly differ in various studies. And there is not yet a universal agreement on the effect of temperature, base fluid, and surfactant on viscosity of TiO_2_ nanofluids. Moreover, the biggest controversy on viscosity of nanofluid is that whether nanofluid is Newtonian fluid or not. The results in Table 3 exhibit that a substantial part of TiO_2_ nanofluids in their work are Newtonian fluids, but also, some others exhibit non-Newtonian behavior. The pronounced differences in different samples are mainly due to the complex influence factors on the rheological property. The shear rate has been proved to have great effect on the rheological property, and also, it has combined effect with other factors including temperature, shearing time, particle loading, base fluid type, and particle shape [124], which make it rather difficult to predict whether a nanofluid is Newtonian fluid or not except by experimental means.

### Surface Tension of Nanofluids

The research on surface tension of TiO_2_–H_2_O nanofluids is much less than that of thermal conductivity or viscosity. Some results showed that adding TiO_2_ nanoparticles had little effect on the surface tension of nanofluids. Liu et al. [125] prepared TiO_2_–H_2_O nanofluids whose particle size ranged from 11 to 50 nm and the surface tensions TiO_2_–H_2_O nanofluids were investigated experimentally. They found the surface tension had no obvious change with the increase in particle loading because the surface tension of nanofluids (1% mass fraction) increased only 1.6% compared with deionized water. Hu et al. [126] found the surface tension of TiO_2_–H_2_O nanofluids increases slightly when adding nanoparticles. And the surface tension decreased as an increase in temperature. Buschmann and Franzke [127] found that no obvious variation occurs when adding a high-volume fraction (5 vol.%) of TiO_2_ nanoparticles in water. Tian and Wang [128] measured the surface tension of TiO_2_–water nanofluids by Jolly balance and abruption method. They found that the surface tension behavior of TiO_2_–water nanofluid was the same as water viz. the surface tension decreased as the temperature increases. However, the variation of surface tension is related to the content of nanoparticles. When the content of nanoparticles increases rapidly, the decrease rate of surface tension of TiO_2_–water nanofluids will slow down. Yang et al. [129] observed that nanoparticles have little effect but the surfactant can greatly change the surface tension of nanofluids, when the loading of surfactant is below the critical micelle concentration (CMC). And they explained this appearance as follows: The effect of surfactant on the surface tension of liquid is much greater than that of nanoparticles. When adding nanoparticles into a fluid containing surfactant whose loading is below CMC, the “free” surfactant will be absorbed on the surface of nanoparticles and then immersed in the liquid, which can weaken the reducing effect of surfactant on the surface tension of liquids.

**Fig. 14:**
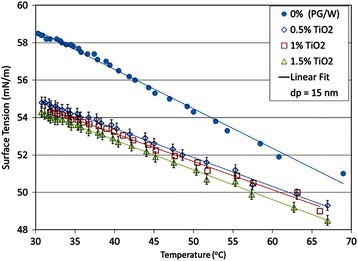
Variation of measured surface tension values of the TiO_2_ nanofluids with temperature [130]. For different volumetric concentrations up to 1.5% and containing 15 nm particles. Reproduced with permission from Elsevier

However, some results also revealed that the nanoparticles played an indispensable role in the surface tension of nanofluids. Chinnam et al. [130] measured the surface tensions of Al_2_O_3_, ZnO, TiO_2_, and SiO_2_ nanofluids with a mixture of 60% propylene glycol and 40% water as base fluids, respectively. They only used one average particle size of 15 nm for TiO_2_ nanofluid due to limiting of manufacturer. They presented a single correlation as a function of volume loading and particle size as well as temperature for all the nanofluids by statistical analysis based on the experimental results. The experimental and fitting results related to TiO_2_ nanofluids are shown in Fig. 14. It was observed that the surface tension of nanofluids decreased as the temperature and particle volume loading increase and the correlation perfectly fitted the experimental data. In addition, they also observed that the surface tension decreased as the particle size decrease for a certain loading and temperature of nanofluids except the ZnO nanofluid.

Although the surface tension study of nanofluid is not as prevalent as studies in thermal conductivity or viscosity, surface tension is also an important parameter which can affect the film flow especially the initial infiltration of film and the probability of forming channel flow. Due to the effect of surfactant on surface tension of nanofluids is greater than nanoparticles, some researchers thought that the reduction in surface tension by surfactant SDBS can produce a superior enhancement of pool boiling performance in R141b-based nano-refrigerant [131].

## Conclusions

The first part of the review focuses on the preparation and two properties viz. viscosity and surface tension of TiO_2_ nanofluids. It can be concluded that although one-step method is expected to achieve better dispersion stability, the side effects of the one-step method such as producing by-product and requiring special solution environment seem more fatal because they severely restrict the application scope of nanofluids. Suitable treatments such as adding dispersant, adjusting pH values, and physical means (stirring and sonication) used singly or in combination can greatly improve the dispersion stability. And the two-step method is recommended to be employed with appropriate post-treatment for the preparation of TiO_2_ nanofluids.

Particle loading is positively correlated to the viscosity, but the effects of other factors are not unified. The viscosities greatly differ in different researches which make the viscosity models hard to predict the experimental value, and hence, the experimental mean is firstly recommended. The surface tension of TiO_2_ nanofluids is more sensitive to surfactant than nanoparticles. The surfactant with low concentration is suggested to be used when it not brings obvious increase in viscosity and foaming ability due to the potential advantages such as reduction in surface tension and improvement in re-dispersible property.
